# Apatinib inhibits VEGF signaling and promotes apoptosis in intrahepatic cholangiocarcinoma

**DOI:** 10.18632/oncotarget.7948

**Published:** 2016-03-07

**Authors:** Hong Peng, Qiuyang Zhang, Jiali Li, Ning Zhang, Yunpeng Hua, Lixia Xu, Yubin Deng, Jiaming Lai, Zhenwei Peng, Baogang Peng, Minhu Chen, Sui Peng, Ming Kuang

**Affiliations:** ^1^ Department of Liver Surgery, The First Affiliated Hospital of Sun Yat-sen University, Guangzhou, Guangdong, China; ^2^ Department of Internal Medicine, The University of Texas Southwestern Medical School, Dallas, Texas, USA; ^3^ Department of Gastroenterology and Hepatology, The First Affiliated Hospital of Sun Yat-sen University, Guangzhou, Guangdong, China; ^4^ Laboratory of Research Center for Translational Medicine, The First Affiliated Hospital of Sun Yat-sen University, Guangzhou, Guangdong, China; ^5^ Department of Pancreato-Billary Surgery, The First Affiliated Hospital of Sun Yat-sen University, Guangzhou, Guangdong, China; ^6^ Department of Oncology, The First Affiliated Hospital of Sun Yat-sen University, Guangzhou, Guangdong, China; ^7^ Department of Medical Ultrasound, Division of Interventional Ultrasound, The First Affiliated Hospital of Sun Yat-sen University, Guangzhou, Guangdong, China

**Keywords:** intrahepatic cholangiocarcinoma, VEGFR2, apatinib, apoptosis, PI3K

## Abstract

Tumor cells co-express vascular endothelial growth factor (VEGF) and VEGF receptors (VEGFRs) that interact each other to support a self-sustainable cell growth. So far, this autocrine VEGF loop is not reported in human intrahepatic cholangiocarcinoma (ICC). Apatinib is a highly selective VEGFR2 inhibitor, but its effects on ICC have not been investigated. In this study, we reported that VEGF and phosphorylated VEGFR2 were expressed at a significantly high level in ICC patient tissues (*P*<0.05). *In vitro*, treating ICC cell lines RBE and SSP25 with recombinant human VEGF (rhVEGF) induced phosphorylation of VEGFR1 (pVEGFR1) and VEGFR2 (pVEGFR2); however, only the VEGFR2 played a role in the anti-apoptotic cell growth through activating a PI3K-AKT-mTOR anti-apoptotic signaling pathway which generated more VEGF to enter this autocrine loop. Apatinib inhibited the anti-apoptosis induced by VEGF signaling, and promoted cell death *in vitro.* In addition, Apatinib treatment delayed xenograft tumor growth *in vivo*. In conclusion, the autocrine VEGF/VEGFR2 signaling promotes ICC cell survival. Apatinib inhibits anti-apoptotic cell growth through suppressing the autocrine VEGF signaling, supporting a potential role for using Apatinib in the treatment of ICC.

## INTRODUCTION

Intrahepatic cholangiocarcinoma (ICC), which occurs frequently in Western countries and Southeast Asia, is the second most common type of primary hepatobiliary cancer. Mortality rate of ICC has risen steeply and steadily since the mid 1990s [1]. The prognosis of ICC is generally poor with a five-year overall survival rate of less than 5%, which has remained unchanged in the past 30 years [2]. This poor survival rate is mainly attributed to late diagnosis and lack of effective non-surgical therapeutic modalities for use in this tumor. Searching better drugs for this lethal tumor is in urgent need.

In the last decade, due to the discovery of important role of VEGF and VEGFR in carcinogenesis [3–5], therapeutic strategies against these targets have been widely studied. In biliary tract cancer (BTC), there are conflicting reports regarding the efficacy even in the same drug targeted against VEGF signaling pathway [6]. In current clinical practice, the same protocols have been used to treat BTC patients of the three anatomical cancer types: ICC, extrahepatic bile duct cancer and gallbladder carcinomas. However, recent studies suggest that the three different BTCs vary not only in clinical features and prognostic factors but also in their pathogenesis and molecular expression profiles [7–9], suggesting that patient selection based on tumor biology and molecular markers is critical for effective evaluation of targeted therapies in this disease.

Apatinib (YN968D1) is a novel and highly selective inhibitor of VEGFR2 tyrosine kinase, with a binding affinity 10 times more than that of sorafenib [10]. By a phase III clinical trial, Apatinib has proven to be the only effective drug to the terminal gastric cancer patients who have no chemotherapy indications [11]. Although Apatinib has shown promising therapeutic effects against diverse tumor types in several phase II clinical trials [11–13], our knowledge about the molecular mechanism of this drug is still limited and it remains unknown if Apatinib has an antitumor effect in human cholangiocarcinoma.

In addition to the well-known effects of VEGF in angiogenesis, recent data suggest that autocrine VEGF signaling in tumor cells plays an important role in promoting their proliferation and inhibiting apoptosis. Many different human tumor types have been found to co-express VEGF and its receptors [14–16], but there is no report about their expression pattern in ICC. In this study, we examined the expression of VEGF and VEGFRs in human ICC tissues, investigated the role and mechanism of autocrine VEGF on the anti-apoptotic cell growth and evaluated the inhibitory effects of Apatinib on ICC cell growth.

## RESULTS

### VEGF and pVEGFR2 overexpressed in intrahepatic cholangiocarcinoma tissues

**Figure 1:**
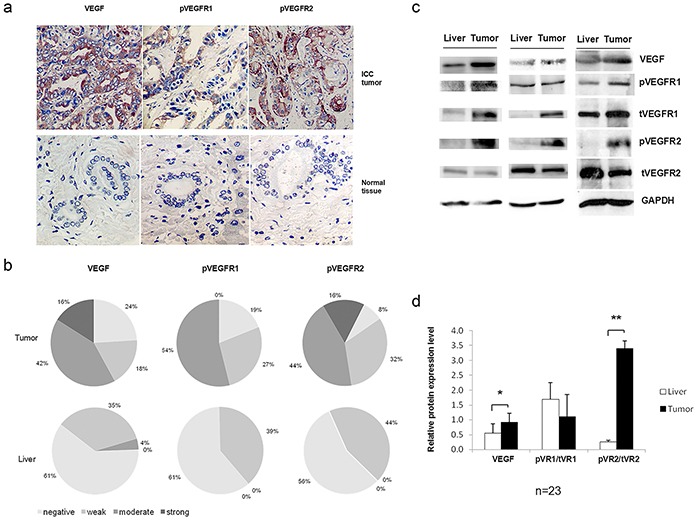
VEGF signaling was activated in intrahepatic cholangiocarcinoma (ICC) tissues **a.** Immunohistochemical staining of VEGF, phospho (p)-VEGFR1, and pVEGFR2. Representative photomicrographs showing the positive staining of VEGF, pVEGFR1, and pVEGFR2 in the ICC cancer cells (400x). **b.** Comparative analysis of VEGF, pVEGFR1, and pVEGFR2 expression in ICC and normal liver. **c.** & **d.** Western blot analysis of VEGF, and pVEGFR1 and pVEGFR2 expression in fresh ICC tumor (T) and normal liver (L) tissues. GAPDH was included as loading control.

Immunohistochemical (IHC) staining was performed to investigate the expression of VEGF and VEGFR2 in104 ICC patients (N=104). The results showed VEGF was located in the cell membrane and cytoplasm of the ICC cells (Figure 1a). Sixteen percent of ICC tissue showed strong VEGF staining and 42% showed moderate staining. Only 4% of the normal liver cells had moderate staining and no strong staining was observed in the normal liver (Figure 1b). It was known that VEGF receptor (VEGFR) relocated to cytoplasm and nuclei when it is activated and becomes phosphorylated [17]. The IHC staining pattern of pVEGFR1 and pVEGFR2 was different (Figure 1a). pVEGFR1 was mainly located in the cytoplasm of ICC cells, while pVEGFR2 was located on the cell membrane, cytoplasm and nuclei of the ICC cells (Figure 1a). In this experiment, 54% of the ICC tissues showed moderate pVEGFR1 staining and no strong staining was seen (Figure 1b). Figure 1a showed the VEGF, pVEGFR1 and pVEGFR2 basal expression level and distribution in ICC cells and normal tissues. In the left panel, the VEGF staining pattern indicated the VEGF located on cytoplasm and nuclei in ICC cells. In the middle panel, the pVEGFR1 staining pattern showed pVEGFR1 mainly located on the cytoplasm in ICC cells. pVEGFR2 located on membrane, cytoplasm and nuclei of the ICC cells as shown in the right panel. The staining pattern of pVEGFR2 was different with pVEGFR1. There is stronger staining of pVEGFR2 on the membrane and nuclei. Sixteen percent of the ICC tissues stained strong and 44% stained moderated for pVEGFR2 (Figure 1b). In contrast, no strong or moderated staining for pVEGFR1 and pVEGFR2 was observed in normal liver cells (Figure 1b).

To confirm the IHC findings, we performed immunoblots using frozen tumor tissues with matched normal tissue controls from twenty-three of the above patients (N=23). The VEGF protein level was about 80% higher than that of normal livers (*P*<0.05) (Figure 1c and 1d). Although the ratio of pVEGFR1/tVEGFR1 was not different between ICC and normal liver, the ratio of pVEGFR2/tVEGFR2 was 13.6 folds higher than that of normal liver (*P*<0.01) (Figure 1c and 1d).

### VEGF activated VEGF receptors and inhibited apoptosis in ICC

**Figure 2:**
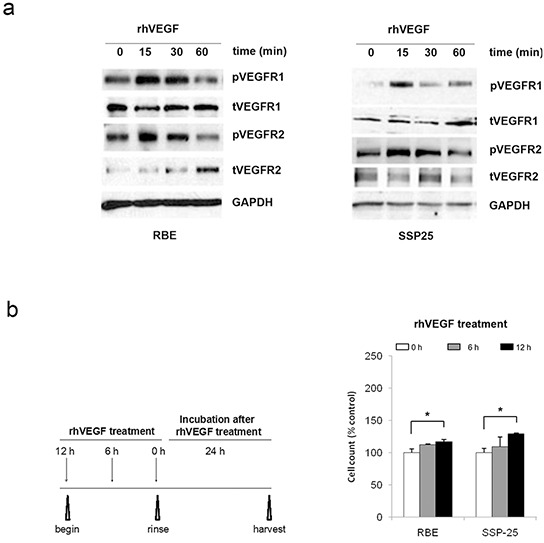
VEGF signaling promoted cell growth in RBE and SSP25 cells Increased phosphorylation of the VEGFR1 and VEGFR2 proteins **a.** and cell growth **b.** in response to recombinant human VEGF (rhVEGF) treatment. Total protein was analyzed by Western blotting with GAPDH included as a loading control. Cell growth was determined by counting the cell number.

Knowing that VEGF expression and signaling activity was significantly higher in ICC tissues, we next determined the role of VEGF signaling in tumor cell growth *in vitro*. We selected two ICC cell lines RBE and SSP25 which all expressed VEGF and activated VEGF receptors pVEGFR1 and pVEGFR2 (Supplementary Figure S1). To determine if the VEGF receptors are active in ICC cells, we treated these two cells with recombinant human (rh) VEGF and examined the level of receptor phosphorylation after different time intervals. In RBE and SSP25 cells, the level of pVEGFR1 and pVEGFR2 increased at 15 min after treatment and then gradually declined to basal level (Figure 2a).

**Figure 3:**
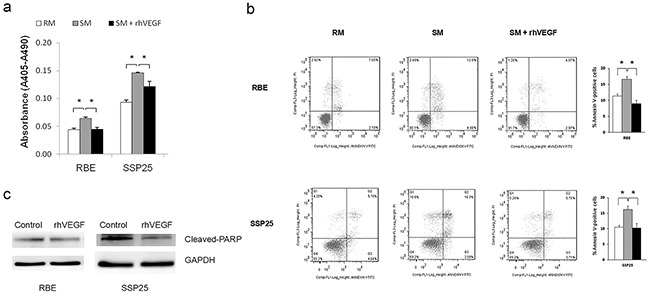
VEGF signaling inhibited apoptosis in RBE, and SSP25 cells The cell apoptosis was induced by switching cells from regular medium (RM) into starvation medium (SM). Cell apoptosis was determined by **a.** ELISA assay **b.** or Annexin V-flow cytometric method. Mean ±standard error of the mean, t-test, *P<0.05, ** P<0.01. **c.** rhVEGF treatment downregulated Cleaved-PARP.

Next, we determined the consequence of VEGFR1 and VEGFR2 activation on cell growth. Treatment with rhVEGF for 6-hr or 12-hr caused a gradual increase of cell counts that had reached a significant level at 24-hr after treatment in all two cell lines (Figure 2b) (*P*<0.05). To explore the mechanism of growth-promoting effect of VEGF/VEGFR signaling, we investigated the cell proliferation and apoptosis. By cell death ELISA, we found that the incubation with starvation medium (SM) significantly induced apoptosis in RBE and SSP25 cells and this increase of apoptosis was significantly suppressed by adding rhVEGF in the SM (Figure 3a) (*P*<0.05). We then repeated these experiments and analyzed cell apoptosis analysis using Annexin V-staining and flow cytometry. Our results confirmed that rhVEGF treatment indeed diminished the apoptosis induced by SM (Figure 3b) (*P*<0.05). On the other hand, the rhVEGF treatment did not significantly enhance cell proliferation (measured by BrdU incorporation) in both cell lines (Supplementary Figure S2). This result indicated that VEGF and VEGFR promoted tumor growth by inhibiting apoptosis in tumor cells. We further performed Western blot cleaved PARP, a terminal effectors related to cell apoptosis and confirmed the result by Annexin V-staining (Figure 3c).

### VEGFR2 played an essential role on VEGF-mediated anti-apoptosis

**Figure 4:**
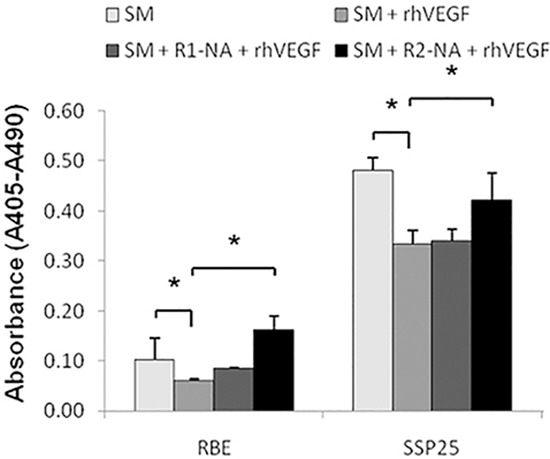
The VEGFR2 played an essential role on anti-apoptotic cell growth in ICC Analysis of ICC cell apoptosis by ELISA. SM=starvation medium, rhVEGF-recobinant human VEGF, R1-NA=VEGFR1 neutralizing antibody, R2-NA=VEGFR2 neutralizing antibody.

The above results promoted us to determine the role of each VEGFR1 and VEGFR2 on cell apoptosis. The rhVEGF inhibited apoptosis induced by SM (Figure 4a) (*P*<0.05). Blocking VEGFR1 did not significantly reverse the effect of rhVEGF treatment (Figure 4a). On the other hand, blocking VEGFR2 significantly reversed the anti-apoptotic effects induced by rhVEGF treatment (Figure 4a) (*P*<0.05). This result indicated that it is the VEGFR2, not VEGFR1 that mediated the effect of VEGF.

### VEGF inhibited apoptosis through VEGFR2/PI3K/Akt/mTOR pathway

**Figure 5:**
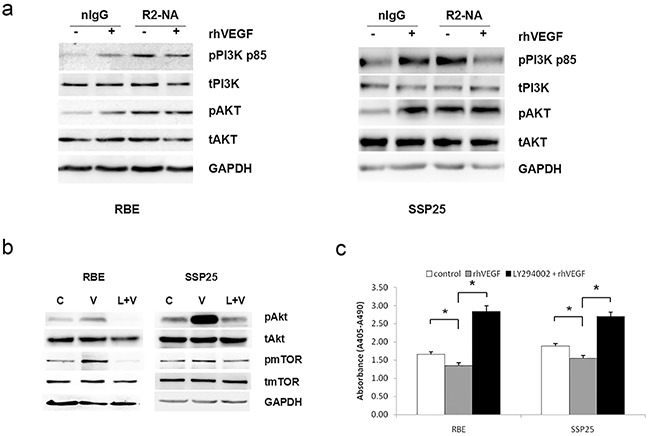
VEGF signaling through a phosphoinositide-3-kinase (PI3K)/protein kinase B(Akt)-dependent pathway in ICC cells **a.** The expression of phosphor (p)-PI3K and pAkt in response to recombinant human VEGF (rhVEGF) treatment in the absence or presence of VEGF receptor inhibitor. R1-NA=VEGFR1 neutralizing antibody, R2-NA=VEGFR2 neutralizing antibody. **b.** & **c.** The essential role of PI3K on the VEGF signaling in ICC cells. The expression of the pAkt and pmTOR proteins (b) and apoptotic cell death (c) in RBE and SSP25 cells after incubation with a PI3K inhibitor LY294002. The protein levels were measured by Western blotting and GAPDH was included as a loading control. Cell apoptosis was measured by cell death ELISA. Mean±SEM, t-test, *P<0.05. ** P<0.01, C=control, V=rhVEGF, L=LY294002.

To determine the signaling pathway related to VEGF-mediated anti-apoptosis, we treated RBE and SSP25 cells (which had a similar response to rhVEGF, see Figure 3a) with VEGFR neutralizing antibodies(NA) for 24 h followed by 15-min rhVEGF treatment and examined the expression of pPI3K and pAKT, that are the downstream pathway molecules of VEGFR2. Treatment with rhVEGF increased the phosphorylation of PI3K and AKT proteins in the two cell lines (Figure 5a). After blocking with VEGFR2-NA, the phosphorylation of PI3K and AKT following the rhVEGF treatment were no longer increased or even reduced (Figure 5a), suggesting that VEGFR2 was responsible for activation of PI3K and AKT molecules.

To determine if the PI3K was indeed the intermediate signaling molecule between VEGFR2 and AKT pathway, we exposed the RBE and SSP25 cells to 50mM of LY294002, a pharmacological inhibitor against PI3K, followed by rhVEGF treatment. Again, rhVEGF treatment induced phosphorylation of AKT and increased expression of mTOR protein (downstream molecules of AKT) (Figure 5b). It also suppressed cell apoptosis as determined by cell death ELISA (Figure 5c) and confirmed by Annexin-V staining (Supplementary Figure S3) (*P*<0.05). Inhibition of PI3K diminished these rhVEGF-induced effects (Figure 5b, 5c and Supplementary Figure S3).

### VEGF stimulated a self-sustainable signaling loop in ICC

**Figure 6:**
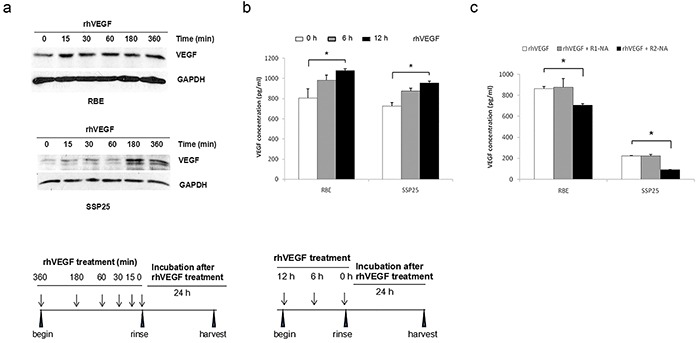
Autocrine VEGF signaling in ICC cells In response to rhVEGF treatment, the intracellular VEGF protein expression **a.** and VEGF secretion **b.** increased in a time-dependent fashion. **c.** The VEGF secretion was downregulated by inhibition of VEGFR2. The intracellular VEGF level was determined by Western blotting, and VEGF secretion by VEGF ELISA. R1-NA=VEGFR1 neutralizing antibody, R2-NA=VEGFR2 neutralizing antibody.

In many human tumors, VEGF stimulation activates a VEGFR2-dependent self-sustainable growth pathway [5, 18]. To explore if similar mechanism exists in ICC cells, we first determined if rhVEGF treatment affects intracellular VEGF protein level. The RBE and SSP25 cell lines were treated with rhVEGF from 15 min to 6 hr. The treatment mediums were then removed and total protein of the treated cells harvested after 24-hr. In RBE, the intracellular VEGF protein was elevated from the 15 min to 6-hr rhVEGF treatment period (Figure 6a). In SSP25, the intracellular VEGF was slightly increased at early time points, and at 3-hr and 6-hr time points, the increase became remarkably (Figure 6a).

We next determined if rhVEGF treatment affected VEGF secretion. After rhVEGF treatment for 6 or 12 hr, the cells were then rinsed with PBS, and incubated in 1% FBS medium for 24-hr. The medium was then collected and the secreted VEGF was measured by ELISA. In both RBE and SSP25 cells, the secreted VEGF gradually increased with increasing rhVEGF treatment time and reached to a significant level at 12-hr (Figure 6b) (*P*<0.05). Blocking VEGFR2, but not VEGFR1, by neutralizing antibody significantly reduced VEGF secretion in these two cell lines (Figure 6c) (*P*<0.05).

### Apatinib treatment increased cell apoptosis by suppressing VEGF signaling

**Figure 7:**
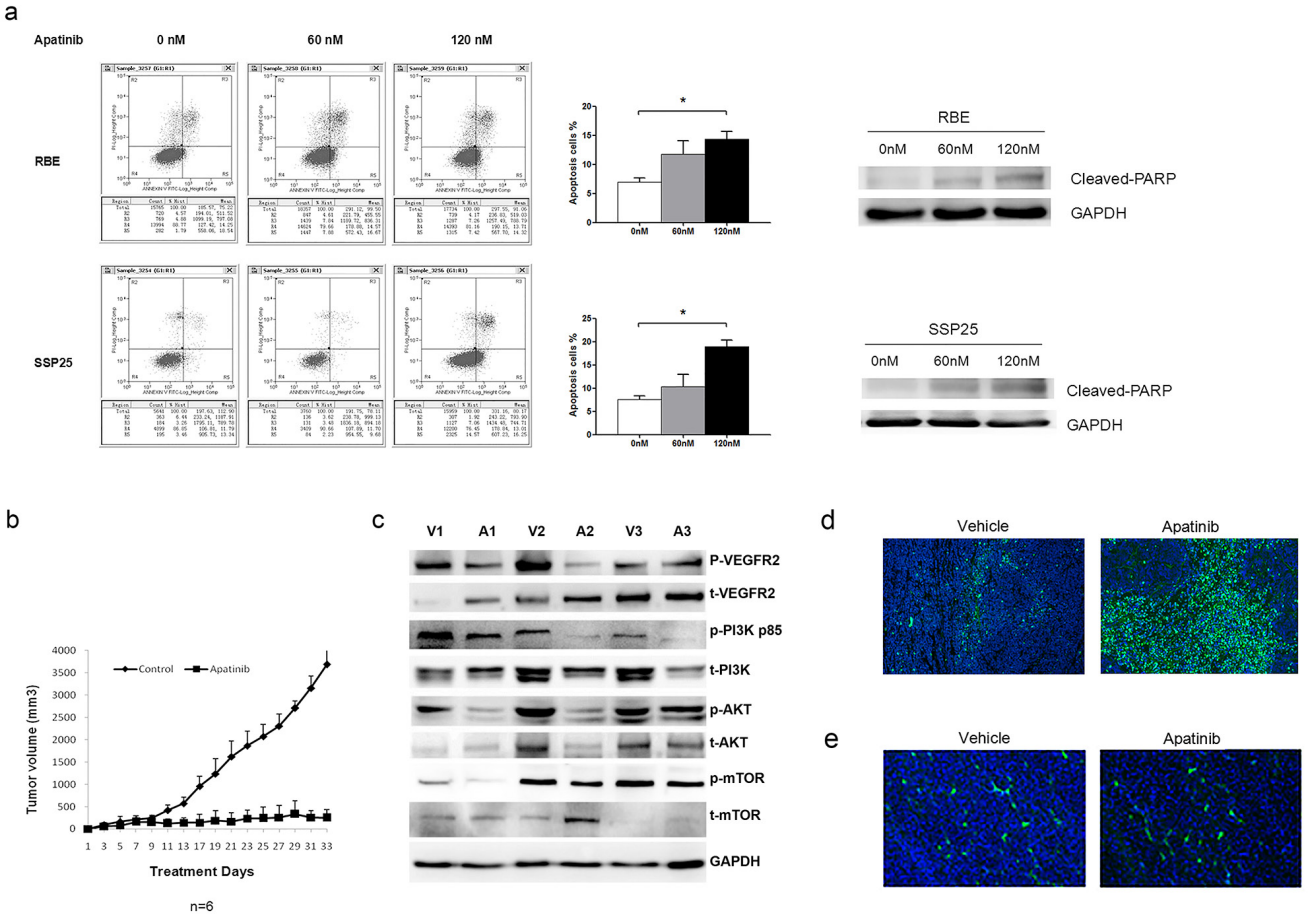
Apatinib suppressed anti-apoptotic cell growth in ICC cells **a.** Apatinib treatment promoted apoptotic cell death in RBE and SSP25 cells. Cell apoptosis was determined by Annexin V-Flow cytometry method and Western Blot method. **b.** Apatinib suppressed RBE tumor growth in xenograft mice. **c.** Apatinib suppressed VEGF signaling in xenograft tumors. The protein levels were measured by Western blotting and GAPDH was included as a loading control. The representative immunoblot was based on tumor tissues from three vehicle (V) and three Apatinib (A)-treated mice. **d.** Representative micrograph showing TUNEL staining of vehicle and Aptinib-treated xenograft tumor (x100). **e.** There was no significant difference of CD31 between the control group and apatinib treatment group.

Apatinib is a small molecule inhibitor that has a potent inhibitory activity against VEGFR2 signaling in lung, colon and stomach cancers [11,19,20]. Treatment with Apatinib at 60 nM and 120 nM resulted in a reduction of pPI3K and pAKT protein level in RBE cells. Treatment with Apatinib at 120 nM resulted in a reduction of pPI3K and pAKT in SSP25 cells (Supplementary Figure S4). The downstream signaling molecules p-mTOR was also inhibited after the treatment of Apatinib. With the same dosages, Apatinib increased cell apoptosis in RBE cells and SSP25 cells (Figure 7a).

**Figure 8:**
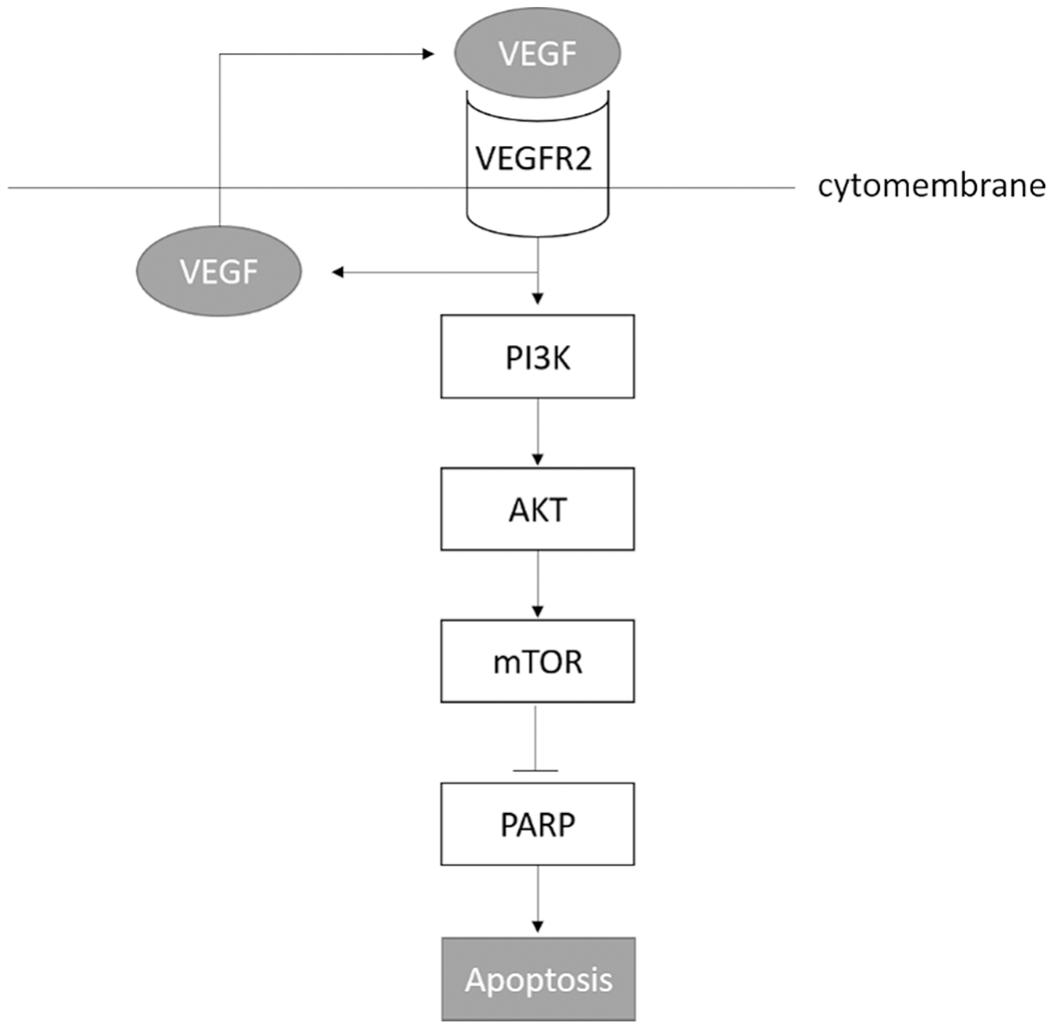
Summary of all findings

We injected RBE cells subcutaneously into NOD/SCID mice. When mice developed a palpable (0.5 cm) mass, they were treated with either Apatinib (50 mg/kg/day) or vehicle solutiondaily until sacrifice (N=6 per group). Compared with vehicle-treated controls, Apatinib-treated mice demonstrated a significant delay in tumor growth (Figure 7b). At sacrifice, the mean total tumor volume for Apatinib-treated mice was significantly lower than that of vehicle-treated control mice (Supplementary Figure S5). Apatinib-treated tumors had reduced expression of pVEGFR2, pPI3K, pAKT and p-mTOR proteins as shown by immunoblots (Figure 7c) and had displayed extensive apoptosis as shown by TUNEL staining (Figure 7d). To identify which effect the tumor volume mostly as shown in figure 7e, we stained CD31 of the tumor and calculated the vessel density between the control group and apatinib treatment group, and found no significant difference of vessel density. We summaries all our findings in Figure 8.

## DISCUSSION

In this study, we found that VEGF and phosphorylated VEGFR2 were overexpressed in ICC tissues. *In vitro*, we demonstrated that VEGF activated VEGFR2 and initiated a PI3K-AKT-mTOR pathway that was both anti-apoptosis and self-sustaining through more VEGF production. We showed that Apatinib inhibited VEGF-VEGFR2-PI3K-AKT signaling, and induced apoptosis in ICC cells. Finally in xenograft mouse model, we showed that treatment with Apatinib induced apoptotic cell death and decreased final tumor volume.

In addition to its well-known angiogenic effect, VEGF may regulate tumor growth through direct binding to VEGFRs present on cancer cells themselves [21]. Although this autocrine VEGF signaling is also reported in normal rat cholangiocytes [22], its role in malignant ICC cells remains unknown. In this study, we found that the activated VEGFR2 (pVEGFR2) was present in cytoplasm as well as in the nuclei of ICC cells. We also found that VEGF could directly induce VEGFR2 phosphorylation leading to the activation of downstream signaling molecules in cell apoptosis pathway. These results suggested that a functional autocrine VEGF loop exists in ICC cells. In addition, we found that inhibition of VEGF -VEGFR2 signaling decreased VEGF production and increased cell apoptosis without significant effect on proliferation. This is consistent with our observations in ICC cells that VEGF activated the pro-survival PI3K-Akt pathway. Recently, Chatterjee *et al* reported that autocrine VEGF/VEGFR2 signaling is required for the initiation of tumor growth *in vivo* [5]. This observation suggested that agents targeted VEGFR2 could be used in the treatment of ICC.

Apatinib is a highly selective inhibitor of VEGFR2 tyrosine kinase. In this study, we found Apatinib has an antitumor effect in human cholangiocarcinoma. The possible mechanism is because Apatinib decreased the VEGF-mediated PI3K/Akt signaling activity and increased cell apoptosis in a dose-dependent manner. Based on these findings, we infer that intracellular VEGFR2 inhibitors, such as Apatinib, have a great potential for use as an anti-tumor agent in ICC patients.

In conclusion, our study demonstrates that the VEGF and VEGFR2 interaction supported ICC cell growth through an angiogenesis-independent anti-apoptotic pathway. The VEGF produced by ICC cells, acts through their own surface receptor VEGFR2 to initiate the downstream PI3K-AKT signaling pathway which resulted in inhibiting apoptosis and enabled cells produce more VEGF to sustain this autocrine cycle. Apatinib inhibits apoptosis both *in vitro* and *in vivo* suggesting that agents targeting molecules involved in autocrine VEGF signaling might be used in the treatment of ICC.

## MATERIALS AND METHODS

### Clinical tissue specimens

Ethical approval for using tissues from human subjects was obtained from the Institutional Review Board of the First Affiliated Hospital of Sun Yat-Sen University (FAHSYSU). Formalin-fixed and paraffin-embedded tissue blocks were archived for use in immunohistochemistry. The specimens were obtained from the ICC patients underwent curative resection in the FAHSYSU. Frozen tissues from twenty-three (N=23) patients were used for immunoblot analysis and written consent was obtained from each patient. The clinical data of 23 ICC patients is shown in Supplementary Table S1.

### Immunohistochemistry

A series of 3-mm sections were obtained from each paraffin block. The sections were subjected to immunohistochemical (IHC) staining procedure for VEGF, phospho (p)-VEGFR1 and pVEGFR2 using an established protocol [18]. The deparaffinized sections were pretreated with 10 mM sodium citrate buffer for antigen unmasking (pH 6.0, boiling temperature, 30 min), blocked in normal serum (Vectastain ABC kit, Vector Laboratories, Inc., Burlingame, CA), incubated with primary antibodies (Supplementary Table S2) at 4°C overnight. Sections were incubated with secondary antibody (Vectastain ABC kit) at room temperature for 30 min. Signals were amplified using Vectastain ABC kit per manufacturer's instruction. Targeted protein was visualized using diaminobenzidine as substrate. The results were interpreted by two independent pathologists (see acknowledgement) who were blinded to the specific diagnosis and prognosis for each case.

### Cell lines

The RBE and SSP25 cell lines were purchased from the Type Culture Collection of the Chinese Academy of Sciences, Shanghai, China. The RBE and SSP25 were grown in RPMI-1640 basal medium with a supplementation of 10% fetal bovine serum and 1 × antibiotics- and 1 × antimycotic solution (Gibco). Cells were maintained in monolayer culture at 37°C in humidified air with 5% CO_2_ in these growth media.

### Total protein extraction and immunoblot analysis

Total protein was extracted using 1X cell lysis buffer for cultured cells according to manufacturer's instruction (Cell Signaling Technology, Danvers, MA). Protein concentrations were determined using a Nanodrop 2000C. Proteins were separated by SDS-polyacrylamide gel electrophoresis, transferred to nitrocellulose membranes and incubated with primary antibodies (Supplementary Table S2) overnight at 4°C. Secondary antibody was either horse anti-rabbit or horse anti-mouse IgG conjugated with horseradish peroxidase (Cell Signaling Technology), and chemiluminescence was determined using a ImageQuant Las4000mini (GE Healthcare, Uppsala, Sweden). The membranes were stripped and re-probed with mouse anti-b-tubulin (Sigma, St. Louis, MO) as a loading control.

### Cell death, cell proliferation, and enzyme-linked immunosorbent assays (ELISA)

Equally seeded wells of cells were incubated in regular medium overnight. Cells were then replaced with an apoptosis-inducing starvation medium (SM) composed of plain medium plus 1% BSA. This step was skipped in the PI3K inhibitor LY294002 (Cell signaling Technology) and Apatinib treatment experiments. After another 16 h, the cells were switched to a treatment medium composed of 1% fetal bovine serum with addition of 30ng/ml of recombinant human VEGF (rhVEGF, R&D systems, Minneapolis, MN), VEGFR1 neutralizing antibody (NA) or VEGFR2-NA (Supplementary Table S2) for another 24 to 48 h. Cell number was determined using a Z1 particle counter (Beckman Coulter, Fullerton, CA). Cell apoptosis was determined by a cell death ELISA kit (Roche, Singapore), and/or by an AnnexinV-flow cytometry method (BD Biosciences, Singapore). Cell proliferation was determined using a BrdU Cell Proliferation Assay kit (Calbiochem, San Diego, CA). The secreted form of VEGF was quantified using a VEGF ELISA kit (Invitrogen, Camarillo, CA). The absorbance of each well was read at a range of wavelengths based on manufacturer's instruction using a TECAN Sunrise plate reader (TECAN Group Ltd, Männedorf, Switzerland).

### Activation/inhibition of VEGF signaling pathway

Cells were equally seeded into 6-well plates. After overnight incubation, cells were starved in 1% FBS medium for 24 hr. Cells were then exposed to medium with 30 ng/ml of rhVEGF for 0, 15, 30, or 60 minutes. Cells were then rinsed twice with PBS, and total proteins were harvested for analysis. To block VEGF signaling, cells were treated with a VEGF neutralizing antibody (VEGF-NA; R&D Systems, Minneapolis, MN), a VEGFR1-NA (Supplementary Table S2), VEGFR2-NA (Supplementary Table S2), a PI3K inhibitorLY294002 (Cell Signaling Technology) or Apatinib (Jiangsu Hengrui Medicine Co., Ltd) before exposing to rhVEGF. In preliminary experiments, cells were treated with Apatinib in doses from 0, 60, 120, 180, 240 nM, and we found that doses ≥ 180 nM of apatinib decreased cell viability. Therefore, 120 nM or less was used for all further experiments.

### Apatinib treatment of xenograft tumors

RBE cells were inoculated at the right flank of NOD/SCID mice. After developing a palpable mass, mice were randomized to either the Apatinib treatment or control group (N=7 per group). Mice were administered a daily oral gavage with 50 mg/kg Apatinib or vehicle-only solution. Tumor size (length and width) was measured every three days and tumor volume was calculated based an established method [23]. At harvest, tumor tissues were processed for Western blotting and TUNEL staining.

### Statistical analysis

The SPSS ver.13.0 (SPSS Inc., Chicago, IL) was used for analysis of the data. The relationship between VEGF, pVEGFR1, pVEGFR2 expression and features of tumor progression were analyzed using the chi-square and the Fisher's exact tests.

## SUPPLEMENTARY FIGURES AND TABLES



## References

[1] Sandhu DS, Roberts LR (2008). Diagnosis and management of cholangiocarcinoma. Curr Gastroenterol Rep.

[2] Wakai T, Shirai Y, Moroda T, Yokoyama N, Hatakeyama K (2005). Impact of ductal resection margin status on long-term survival in patients undergoing resection for extrahepatic cholangiocarcinoma. CANCER-AM CANCER SOC.

[3] Zhang Q, Yu C, Peng S, Xu H, Wright E, Zhang X, Huo X, Cheng E, Pham TH, Asanuma K, Hatanpaa KJ, Rezai D, Wang DH, Sarode V, Melton S, Genta RM (2014). Autocrine VEGF signaling promotes proliferation of neoplastic Barrett's epithelial cells through a PLC-dependent pathway. GASTROENTEROLOGY.

[4] Xia G, Kumar SR, Hawes D, Cai J, Hassanieh L, Groshen S, Zhu S, Masood R, Quinn DI, Broek D, Stein JP, Gill PS (2006). Expression and significance of vascular endothelial growth factor receptor 2 in bladder cancer. J Urol.

[5] Chatterjee S, Heukamp LC, Siobal M, Schottle J, Wieczorek C, Peifer M, Frasca D, Koker M, Konig K, Meder L, Rauh D, Buettner R, Wolf J, Brekken RA, Neumaier B, Christofori G (2013). Tumor VEGF:VEGFR2 autocrine feed-forward loop triggers angiogenesis in lung cancer. J CLIN INVEST.

[6] Ramirez-Merino N, Aix SP, Cortes-Funes H (2013). Chemotherapy for cholangiocarcinoma: An update. World J Gastrointest Oncol.

[7] Park KW, Jung ES, Kim DG, Yoo YK, Hong TH, Lee IS, Koh YH, Kim JH, Lee MA (2013). ERCC1 Can Be a Prognostic Factor in Hilar Cholangiocarcinoma and Extrahepatic Bile Duct Cancer, But Not in Intrahepatic Cholangiocarcinoma. CANCER RES TREAT.

[8] Yoshikawa D, Ojima H, Iwasaki M, Hiraoka N, Kosuge T, Kasai S, Hirohashi S, Shibata T (2008). Clinicopathological and prognostic significance of EGFR, VEGF, and HER2 expression in cholangiocarcinoma. Br J Cancer.

[9] Miller G, Socci ND, Dhall D, D'Angelica M, DeMatteo RP, Allen PJ, Singh B, Fong Y, Blumgart LH, Klimstra DS, Jarnagin WR (2009). Genome wide analysis and clinical correlation of chromosomal and transcriptional mutations in cancers of the biliary tract. J Exp Clin Cancer Res.

[10] Tian S, Quan H, Xie C, Guo H, Lu F, Xu Y, Li J, Lou L (2011). YN968D1 is a novel and selective inhibitor of vascular endothelial growth factor receptor-2 tyrosine kinase with potent activity in vitro and in vivo. CANCER SCI.

[11] Li J, Qin S, Xu J, Guo W, Xiong J, Bai Y, Sun G, Yang Y, Wang L, Xu N, Cheng Y, Wang Z, Zheng L, Tao M, Zhu X, Ji D (2013). Apatinib for chemotherapy-refractory advanced metastatic gastric cancer: results from a randomized, placebo-controlled, parallel-arm, phase II trial. J CLIN ONCOL.

[12] Hu X, Cao J, Hu W, Wu C, Pan Y, Cai L, Tong Z, Wang S, Li J, Wang Z, Wang B, Chen X, Yu H (2014). Multicenter phase II study of apatinib in non-triple-negative metastatic breast cancer. BMC CANCER.

[13] Langer CJ, Mok T, Postmus PE (2013). Targeted agents in the third-/fourth-line treatment of patients with advanced (stage III/IV) non-small cell lung cancer (NSCLC). CANCER TREAT REV.

[14] Hamerlik P, Lathia JD, Rasmussen R, Wu Q, Bartkova J, Lee M, Moudry P, Bartek JJ, Fischer W, Lukas J, Rich JN, Bartek J (2012). Autocrine VEGF-VEGFR2-Neuropilin-1 signaling promotes glioma stem-like cell viability and tumor growth. J EXP MED.

[15] Lee TH, Seng S, Sekine M, Hinton C, Fu Y, Avraham HK, Avraham S (2007). Vascular endothelial growth factor mediates intracrine survival in human breast carcinoma cells through internally expressed VEGFR1/FLT1. PLOS MED.

[16] Adamcic U, Skowronski K, Peters C, Morrison J, Coomber BL (2012). The effect of bevacizumab on human malignant melanoma cells with functional VEGF/VEGFR2 autocrine and intracrine signaling loops. NEOPLASIA.

[17] Stewart M, Turley H, Cook N, Pezzella F, Pillai G, Ogilvie D, Cartlidge S, Paterson D, Copley C, Kendrew J, Barnes C, Harris AL, Gatter KC (2003). The angiogenic receptor KDR is widely distributed in human tissues and tumours and relocates intracellularly on phosphorylation. An immunohistochemical study. HISTOPATHOLOGY.

[18] Peng S, Wang Y, Peng H, Chen D, Shen S, Peng B, Chen M, Lencioni R, Kuang M (2014). Autocrine vascular endothelial growth factor signaling promotes cell proliferation and modulates sorafenib treatment efficacy in hepatocellular carcinoma. HEPATOLOGY.

[19] Li J, Zhao X, Chen L, Guo H, Lv F, Jia K, Yv K, Wang F, Li C, Qian J, Zheng C, Zuo Y (2010). Safety and pharmacokinetics of novel selective vascular endothelial growth factor receptor-2 inhibitor YN968D1 in patients with advanced malignancies. BMC CANCER.

[20] Hu X, Zhang J, Xu B, Jiang Z, Ragaz J, Tong Z, Zhang Q, Wang X, Feng J, Pang D, Fan M, Li J, Wang B, Wang Z, Zhang Q, Sun S (2014). Multicenter phase II study of apatinib, a novel VEGFR inhibitor in heavily pretreated patients with metastatic triple-negative breast cancer. INT J CANCER.

[21] Ferrara N, Gerber HP, LeCouter J (2003). The biology of VEGF and its receptors. NAT MED.

[22] Lichtenberger BM, Tan PK, Niederleithner H, Ferrara N, Petzelbauer P, Sibilia M (2010). Autocrine VEGF signaling synergizes with EGFR in tumor cells to promote epithelial cancer development. CELL.

[23] Holloway SE, Beck AW, Shivakumar L, Shih J, Fleming JB, Brekken RA (2006). Selective blockade of vascular endothelial growth factor receptor 2 with an antibody against tumor-derived vascular endothelial growth factor controls the growth of human pancreatic adenocarcinoma xenografts. ANN SURG ONCOL.

