# Supplementary material for: Apatinib inhibits VEGF signaling and promotes apoptosis in intrahepatic cholangiocarcinoma
Source: Oncotarget. 2016 Mar 7;7(13):17220–9. doi: 10.18632/oncotarget.7948 (PMC4941382; doi:10.18632/oncotarget.7948)
Supplement: Supplementary file 1 [file oncotarget-07-17220-s001.pdf]

SUPPLEMENTARY FIGURES AND TABLES

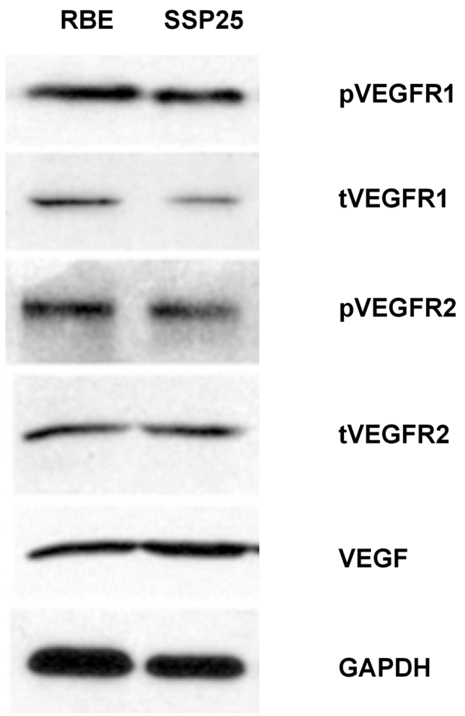

Supplementary Figure S1: Western blotting analysis of basal expression of phospho-(p) -VEGFR1, tVEGFR1, pVEGFR2, and tVEGFR2 and VEGF proteins in RBE and SSP25 cells. GAPDH was included as a loading control.

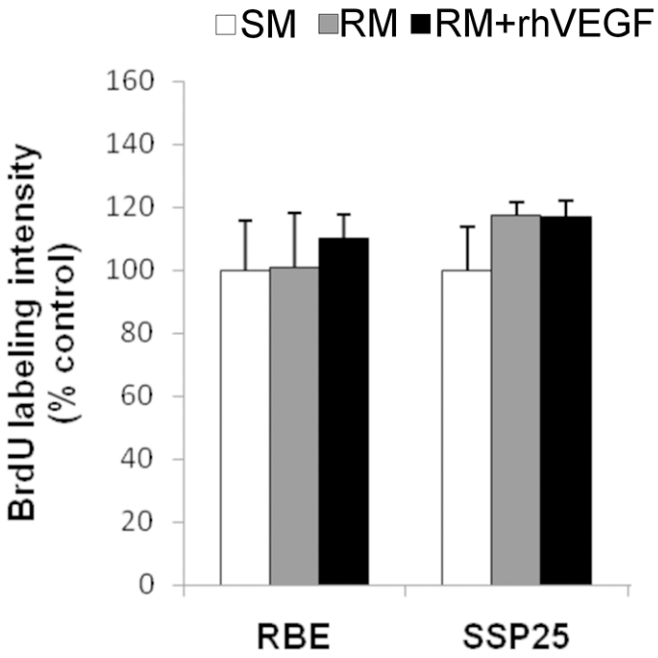

Supplementary Figure S2: Analysis of cell proliferation in RBE and SSP25 cells by BrdU incorporation assays. RM= regular medium, SM=starvation medium, rhVEGF=recombinant human VEGF. Mean±SEM, t-test.

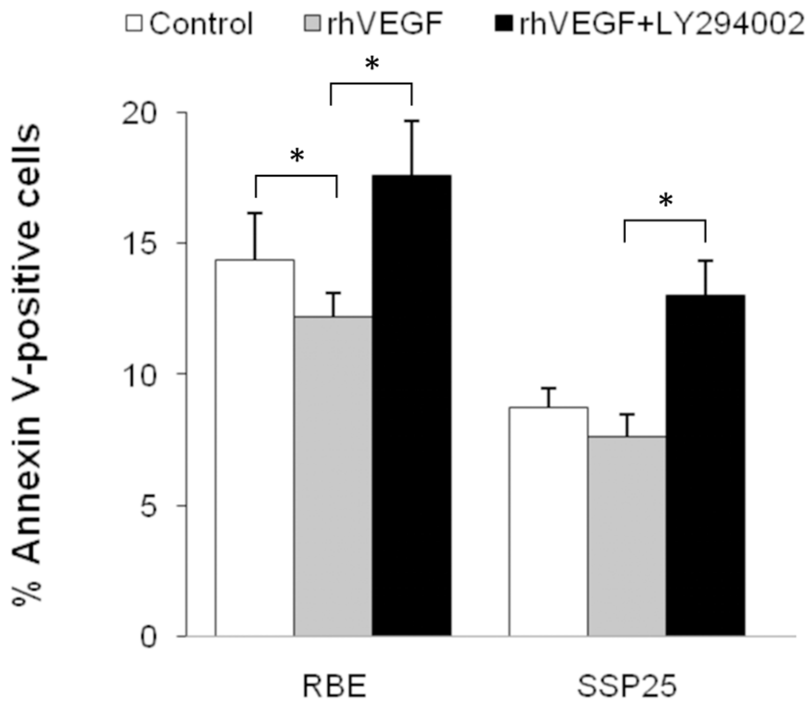

Supplementary Figure S3: The essential role of phosphoinositide-3-kinase (PI3K) on VEGF-mediated anti-apoptotic cell growth in ICC cells. Cell apoptosis was measured by Annexin V staining followed by Flow cytometry.

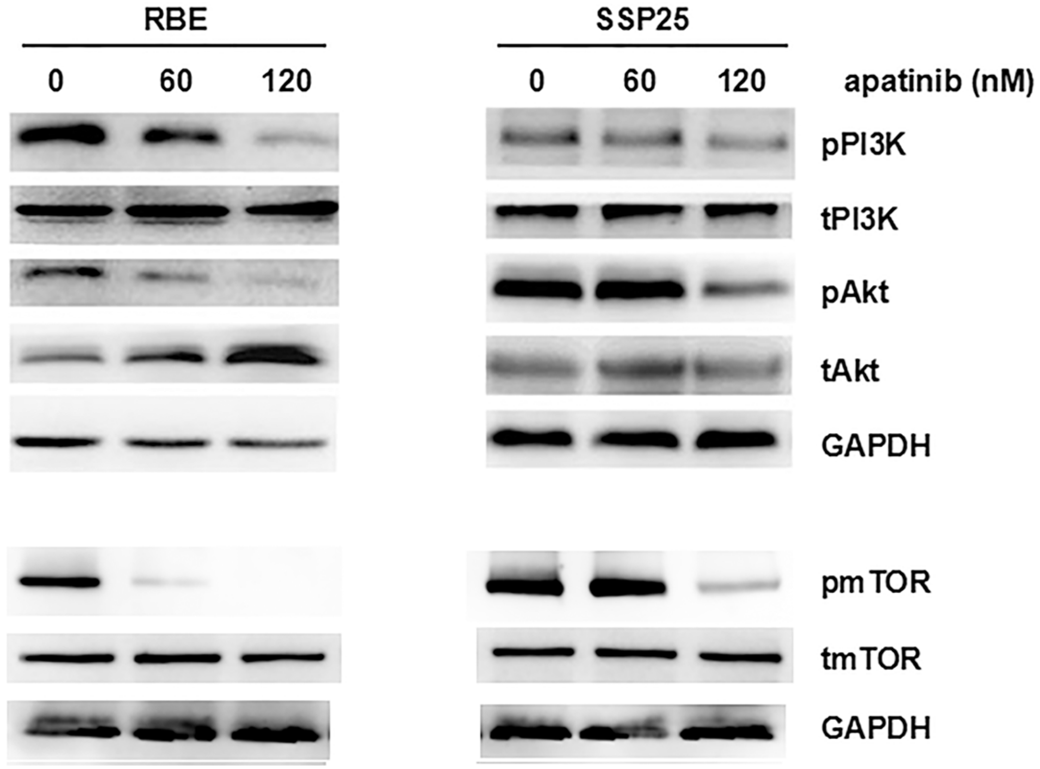

Supplementary Figure S4: Apatinib inhibited the phosphorylation of VEGF pathway molecules PI3K, AKT and mTOR in RBE and SSP28 cells. Total protein was measured by Western blotting with GAPDH as loading control.

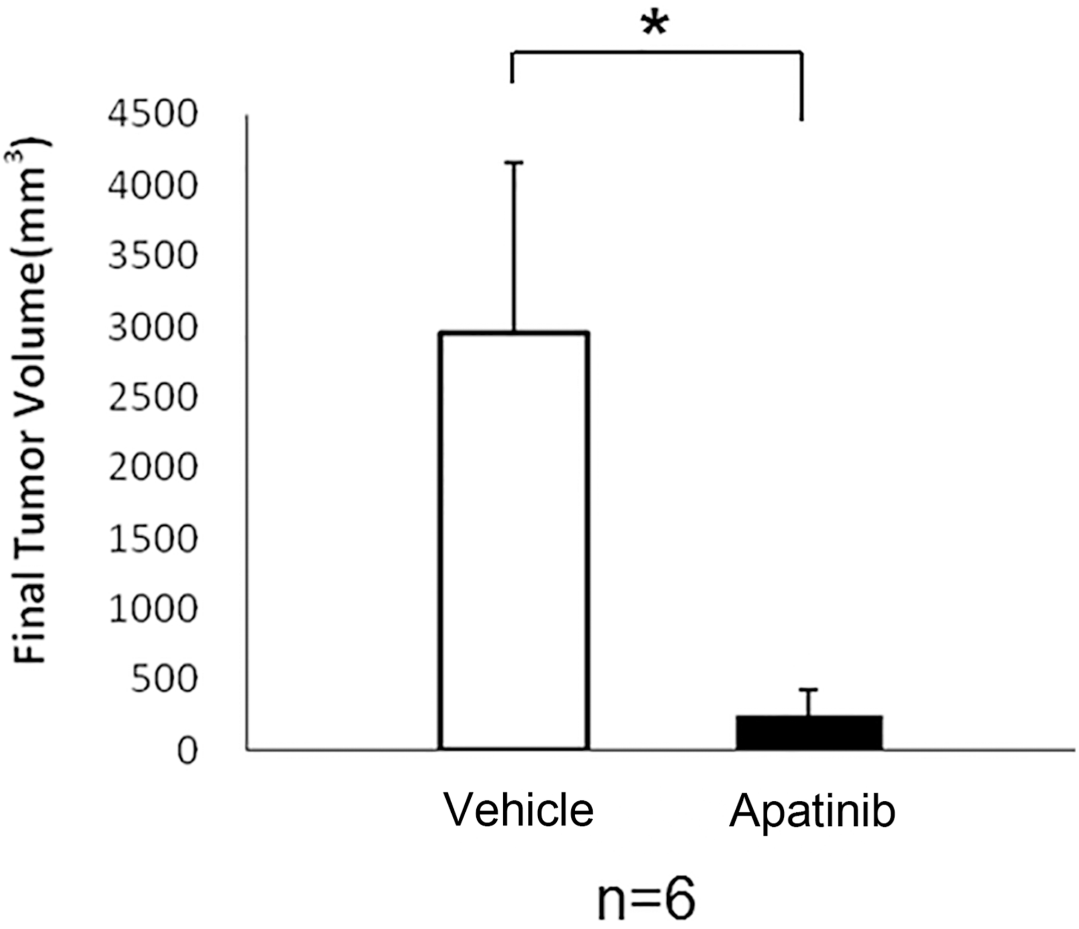

Supplementary Figure S5: Apatinib treatment caused a substantial reduction of final volume of RBE xenograft tumors.

Supplementary Table S1: Clinical data of 23 ICC patients

Variable	Patients (n=23)
Age	54±7.4
Gender	
Male	16
Female	7
Differentiation	
Low	5
Moderate or High	18

Supplementary Table S2: A list of antibodies used in this study

antibody	Source information (cat#, vendor)	dosage	Usage
VEGF	ab1316, Abcam	1:800 dilution	IHC
VEGF	sc-152, Santa Cruz	1 μg/ml	WB
VEGF-NA	AB293-NA, R&D Systems	6 μg/ml	neutralization
VEGFR1-NA	AF321, R&D systems	100 ng/ml	neutralization
VEGFR1	#2893, Cell Signaling	WB, 1:1000 dilution	IHC, WB
pVEGFR1	Ab111835, Abcam	IHC, 1:100	IHC
pVEGFR1	Ab62183, Abcam	1:1000	WB
VEGFR2-NA	MAB3572, R&D Systems	100 ng/ml	neutralization
VEGFR2	#2479, Cell Signaling	1 μg/ml	IF, WB
pVEGFR2	Ab5473, Abcam	IHC, 1 μg/ml; WB, 0.5 μg/ml	IHC, IF, WB
PI3K	#5569, Cell Signaling	0.1 μg/ml	WB
pPI3K	#4228, Cell signaling	0.1 μg/ml	WB
AKT	#9272, Cell signaling	0.1 μg/ml	WB
pAKT	#4060, Cell signaling	0.1 μg/ml	WB
mTOR	#2972, Cell signaling	0.1 μg/ml	WB
pmTOR	#2971, Cell Signaling	0.1 μg/ml	WB
Tubulin	T5293, Sigma	1:3000	WB
GAPDH	MAB374, Millipore	1:3000	WB
normal IgG	sc-2027 L, Santa Cruz	6 μg/ml	Neutralization
		1 μg/ml	IHC
